# Understanding the Osteosarcoma Pathobiology: A Comparative Oncology Approach

**DOI:** 10.3390/vetsci3010003

**Published:** 2016-01-18

**Authors:** Jyotika Varshney, Milcah C. Scott, David A. Largaespada, Subbaya Subramanian

**Affiliations:** 1 Masonic Cancer Center, University of Minnesota, Minneapolis, MN 55455, USA; varsh005@umn.edu (J.V.); scot0340@umn.edu (M.C.S.); larga002@umn.edu (D.A.L.); 2 Department of Surgery, University of Minnesota Medical School, Moos Tower, 11-212420 Delaware Street, S.E.; MMC 195, Minneapolis, MN 55455, USA; 3 Animal Cancer Care and Research Program, University of Minnesota, St. Paul, MN 55455, USA; 4 Department of Veterinary Clinical Sciences, College of Veterinary Medicine, University of Minnesota, St. Paul, MN 55108, USA; 5 Department of Pediatrics, University of Minnesota, Minneapolis, MN 55455, USA

**Keywords:** osteosarcoma, comparative oncology, microRNAs, prognosis, canine osteosarcoma

## Abstract

Osteosarcoma is an aggressive primary bone tumor in humans and is among the most common cancer afflicting dogs. Despite surgical advancements and intensification of chemo- and targeted therapies, the survival outcome for osteosarcoma patients is, as of yet, suboptimal. The presence of metastatic disease at diagnosis or its recurrence after initial therapy is a major factor for the poor outcomes. It is thought that most human and canine patients have at least microscopic metastatic lesions at diagnosis. Osteosarcoma in dogs occurs naturally with greater frequency and shares many biological and clinical similarities with osteosarcoma in humans. From a genetic perspective, osteosarcoma in both humans and dogs is characterized by complex karyotypes with highly variable structural and numerical chromosomal aberrations. Similar molecular abnormalities have been observed in human and canine osteosarcoma. For instance, loss of *TP53* and *RB* regulated pathways are common. While there are several oncogenes that are commonly amplified in both humans and dogs, such as *MYC* and *RAS*, no commonly activated proto-oncogene has been identified that could form the basis for targeted therapies. It remains possible that recurrent aberrant gene expression changes due to gene amplification or epigenetic alterations could be uncovered and these could be used for developing new, targeted therapies. However, the remarkably high genomic complexity of osteosarcoma has precluded their definitive identification. Several advantageous murine models of osteosarcoma have been generated. These include spontaneous and genetically engineered mouse models, including a model based on forward genetics and transposon mutagenesis allowing new genes and genetic pathways to be implicated in osteosarcoma development. The proposition of this review is that careful comparative genomic studies between human, canine and mouse models of osteosarcoma may help identify commonly affected and targetable pathways for alternative therapies for osteosarcoma patients. Translational research may be found through a path that begins in mouse models, and then moves through canine patients, and then human patients.

## 1. Introduction

Osteosarcoma is an aggressive primary bone tumor most prevalent in human patients. It mostly occurs during adolescence, with a second peak at middle age (older than 40) [1,2]. The tumor is characterized by increased production of osteoid (abnormal bone matrix) and by exceptionally complex karyotypes [3,4]. While the target cell for malignant transformation in the formation of osteosarcoma is not known with certainty, it is thought to be a mesenchymal stem cell (MSC) or a cell committed to the osteoblast lineage [5]. Evidence from mouse models indicates that any of several cell stages could serve as target cells for osteosarcoma development [6]. There are three common histologic types of osteosarcoma: osteoblastic, where tumor cells produce large amounts of tumor osteoid; chondroblastic, where tumor cells produce chondroid (cartilage) in addition to some amount of tumor osteoid; and fibroblastic, where tumor cells are predominantly fibroblasts and can produce both collagen and tumor osteoid [7]. The disease is highly metastatic, with distant spread mostly to lungs and other sites in bone, but osteosarcoma can also metastasize to lymph nodes and intra-abdominal organs [8,9]. The metastatic pattern (lungs, bones, lymph nodes) is similar for dogs and humans [10]. Osteosarcoma may present with macroscopic metastatic disease or metastatic disease can occur after therapy. In either case, prognosis is significantly worse once metastasis has been detected [11,12].

In canine patients, osteosarcoma is a disease primarily of adult dogs; the median age at diagnosis is approximately eight years, with a small peak of incidence in young animals (younger than 3 years) [10,13]. This differs from the situation in human patients where the peak age of incidence is in adolescence. Nevertheless, the natural history of the disease is similar in canine and human osteosarcoma patients [14]. Moreover, we have observed that genome wide expression profiles of canine osteosarcoma are indistinguishable from human pediatric osteosarcoma and more like human osteosarcoma than any other human cancer [14,15]. In dogs, there is generally a strong breed preference in the risk for cancer, such as osteosarcoma [16,17]. Many large breed dogs have an increased risk for osteosarcoma compared to other breeds [4,18,19]. The genetic determinants of osteosarcoma susceptibility in dogs are not fully elucidated, and it is possible that these factors will also be important in human osteosarcoma susceptibility. The development of treatment strategies in dogs and people has proven mutually beneficial for both species [20]. In fact, current treatment options are similar in both species.

Osteosarcoma is treated using surgery, radiation and chemotherapy. Surgical techniques have greatly improved with time, and in many cases limb salvage is feasible with low rates of local recurrence in humans [21]. Combination chemotherapies are vital for effective treatment of osteosarcoma [22]. However, despite decades of effort, nothing better than the most often used combination of three chemotherapeutic agents (*i.e.*, methotrexate, doxorubicin and cisplatin) has been found [22].The adjuvant chemotherapy is generally administered prior to surgery and, according to many studies, the extent of necrosis observed in the primary tumor after surgical removal is correlated with the outcome. [21]. The goal of the adjuvant chemotherapy is to eliminate the micrometastases that are thought to be present in >80% of all patients at the time of diagnosis, thus preventing relapse [1]. New research is underway to define specific molecular targets that could be utilized to treat this disease, but their elucidation has been proven difficult. It is possible that osteosarcoma may also be treated using recently developed immunotherapies, such as immune checkpoint blockade or chimeric antigen receptor-engineered T cells [21]. 

Osteosarcoma is characterized by aneuploidy and extensive genetic instability [23]. Due to this extreme genetic instability, common causes of osteosarcoma development have largely been limited to implicating loss of *RB* and *TP53* regulated cellular activities and upregulation of *MYC* transcriptional activity reviewed by Morrow *et al.* [24]. Beyond these, few alterations are common or well accepted as osteosarcoma drivers. Gene amplification and protein overexpression of the *RUNX2* transcription factor, which is a regulator of bone renewal, has been proposed to drive osteosarcoma [25]. Conflicting data on activation of beta-catenin dependent transcription in osteosarcoma has been reported [26]. In addition, TGF-β signaling has been proposed to promote the acquisition of metastatic disease [27]. Notable, expression of Ezrin at high levels has been found in metastatic human and canine osteosarcoma and functionally validated in model systems as a driver of osteosarcoma metastasis [28]. Recently, a large scale *Sleeping Beauty* (SB) transposon-based forward genetic screen was carried out for osteosarcoma in mice [29]. From this screen, a large number of new candidate osteosarcoma proto-oncogene and tumor suppressors were reported. These included the candidate oncogenes *SEMA4D* and *SEMA6D*, which were partially functionally validated as well as tumor suppressors like *NF1*, *NF2* and *PTEN* [29]. Beyond protein encoding genes, several micro RNAs (miRNAs) and miRNA clusters have been suggested as drivers or suppressors of osteosarcoma development and progression [30]. What remains unclear is which common oncogenic drivers for osteosarcoma should be targeted and how they would be targeted. Unlike the case for other malignancies, osteosarcoma is genetically heterogeneous and common molecular targets may not be present.

Osteosarcoma is much more common in dogs than in people (greater than 15 times) and can be experimentally induced in mice via transgenesis. Perceivably, the opportunity to study osteosarcoma in three different species may make it possible to identify core genes and pathways, whose alterations are central to the osteosarcoma phenotype and that would make good targets for therapy. In the past, the main challenges were the development of improved surgical techniques for limb salvage, prevention of local recurrence and the identification of effective combination chemotherapies. Currently, a major pressing need is the development of new therapies to prevent the outgrowth of metastatic disease and to treat it once it has occurred. It is possible that germline or somatic mutations or tumor gene expression signatures identified through multi-species approaches could help predict which patients will respond well to current treatment regimes and which need to be referred for more intensive therapy or follow-up screening. However, much remains to be learned about what allows microscopic osteosarcoma lesions to resist chemotherapy and to suddenly break tumor dormancy and form a macroscopic mass. Until these questions are answered, we are not fully capable to help many human and canine osteosarcoma patients.

This review is an attempt to address many of the unanswered questions pertaining to osteosarcoma by focusing on recently acquired knowledge from a multi-species approach.

## 2. Human Osteosarcoma

Osteosarcoma in human patients is a rare tumor with a peak incidence in the second decade of life. The incidence is roughly 25 cases per 10 million per year in the United States [31]. A second wave of osteosarcoma is diagnosed in individuals between 70 and 80 years of age, often in association with Paget’s disease. Paget’s disease is an abnormality of bone homeostasis in which excessive bone reabsorption and reformation occur. Genetic and viral origins of Paget’s disease have been suggested. While osteosarcoma is a rare manifestation of the disease, risk for osteosarcoma is increased. Osteosarcoma can occur in nearly any part of the skeleton, but commonly affects one of the long bones. As it occurs during a period of intense bone growth, there may be an association between this growth and sensitivity to osteoblast transformation [31]. Indeed, children with osteosarcoma are on average tall for their age [32]. Genetic studies also have suggested an association between the inheritance of certain forms of growth control genes and risk for osteosarcoma [33]. Other risk factors for human osteosarcoma include prior radiation therapy, male gender, and possibly African ancestry [34,35]. A genome-wide association study (GWAS) revealed possible risk alleles in the glutamate receptor, metaotropic 4 (*GRM4*) gene and a gene desert at chromosome 2p25.2 [36]. A recent GWAS also identified a potential high-risk allele for osteosarcoma metastasis at presentation within the transcriptional activator *NFIB* [37], a gene that was also identified as a potential tumor/metastasis suppressor in a transposon-based genetic screen in mice [29].

Some rare inherited cancer predisposition syndromes increase the risk for osteosarcoma. These rare cancers include Li-Fraumeni syndrome, hereditary retinoblastoma and Rothman-Thompson syndrome caused by germline mutations in *TP53, RB1*, and *RECQL4*, respectively [38]. Certain other cancer predisposition syndromes also seem to increase the risk of osteosarcoma development including Neurofibromatosis Type 1 syndrome, Werner’s syndrome and Diamond-Blackfan anemia [24]. Patients with hereditary multiple osteochondromas, due to inheritance of mutations in the *EXT1* or *EXT2* genes, are predisposed to development of osteosarcoma also [24].

Human osteosarcoma is typically diagnosed due to swelling and pain often in the limbs. This can cause a limp if the involved limb is a leg or immobility of a joint. Pain may be constant or associated with exertion. In rare cases osteosarcoma will trigger a bone fracture. Diagnosis is made following a complete medical exam and imaging studies. Often an X-ray is done, followed by positron emission tomography or a bone scan. As osteosarcoma can often metastasize to the lungs, lung X-rays are utilized. A definitive diagnosis requires a biopsy. Staging of osteosarcoma is controversial. Most believe that all osteosarcoma should be considered high grade and treated as such although low grade osteosarcoma seems to be a distinct entity [38]. The only commonly accepted staging is localized *versus* metastatic disease.

Human osteosarcoma is commonly treated using surgery and chemotherapy [38]. Chemotherapy for roughly ten weeks that precedes surgery and the extent of necrosis in the surgically removed primary tumor has been suggested to be associated with long term risk for disease recurrence [38]. Chemotherapy is also given after surgery for a period of up to a year. Usually two or three chemotherapeutic agents are given for osteosarcoma treatment. Common combinations include high-dose methotrexate, doxorubicin and cisplatin (sometimes with ifosfamide); doxorubicin and cisplatin; ifosfamide and etoposide; ifosfamide, cisplatin (or carboplatin) and epirubicin [38]. Radiation therapy is used in some cases for non-resectable tumors [38].

The prognosis of human osteosarcoma is affected largely by the presence or absence of metastases. The 5-year survival rate for localized osteosarcoma is estimated to be between 50% and 60%. For metastatic disease the 5-year survival rate is 15% to 30% [39]. There is a better prognosis if metastatic osteosarcoma is present only in the lungs and is all metastatic lesions can be removed surgically [39]. That said, there is a dire need to develop therapies that can be used to treat patients who present with extensive metastatic disease or who recur with metastatic disease. At present, palliative care is often used in such cases or referral for clinical trials [38].

## 3. Canine Osteosarcoma

Canine osteosarcoma accounts for 80%–90% of canine primary bone tumors [40]. Dogs often present with a history of lameness or in some cases with a pathologic fracture of the affected bone. Diagnosis is based on clinical signs, imaging and biopsy. The metaphyseal region of long bones is the most common primary site with front limbs affected twice as often as rear limbs and the distal radius and proximal humerus being the two most common locations [41,42].

It is believed that external factors such as chemical carcinogens, ionization radiation, metallic implants to fix fractures and precedent skeletal disorders such as osteomyelitis and microscopic fractures can lead to canine osteosarcoma [10]. There are implications of genetic factors such as *TP53* and RB1 aberrations, as well as certain viruses and growth factor alterations [43]. Osteosarcoma can affect any breed of dog, but it is more commonly found in the larger breeds. Some breeds, such as the Scottish Deerhound, Great Dane, St. Bernard and Greyhound, are at high risk for developing primary bone tumors suggesting a genetic predilection [40]. In fact, the CDKN2A locus has been recently linked to osteosarcoma risk in dogs, and the risk allele is fixed in certain breeds like Rottweilers and Irish Wolfhounds [4].

Without treatment, the estimated survival for a dog with osteosarcoma is less than 3 months. On the other hand, survival times of approximately 1 year (or about 10% of a dog’s lifetime) are achievable for 20%–50% of dogs with osteosarcoma treated using the current standard of care, and a small percentage of dogs can survive up to 5–6 years after diagnosis [44]. Standard of care for dogs is surgery (amputation of limb sparing surgery) with adjuvant chemotherapy [45]. The choice of chemotherapy drugs does not seem to influence survival, thus, toxicity, quality of life and cost tend to be the factors that guide treatment decisions [44]. Chemotherapy is only recommended when the primary tumor is removed and as of current, the drug of choice is carboplatin.

Many well-controlled studies show that a clinical response for osteosarcoma is only achievable with accepted standard of care. In very rare cases, dogs with osteosarcoma that receive palliative care may have prolonged (>1 year) survival, even in face of metastatic disease. Anecdotal benefits reported from herbal or “alternative” treatments, have not been reproducible, and no alternative therapies have been shown to have efficacy or provide consistent clinical benefit in controlled trials.

Metastatic bone cancer is the common cause of death or euthanasia, in 90% of dogs by 1 year [46]. Reducing the primary tumor’s ability to metastasize and enhancing the antitumor activity of chemotherapy drugs and yet having minimal negative side effects is still a challenge for veterinary practitioners [10]. Treatment options for canine patients with metastases include pulmonary metastasectomy, but treatment for metastatic disease is only recommended if the primary tumor remains in complete remission [47]. The median survival after pulmonary metastasectomy can be up to 6 months; but without standard surgical procedure, survival outcome is usually less than 2 months [46].

## 4. Mouse Models of Osteosarcoma

The number of genetically engineered or other mouse models of osteosarcoma are fairly restricted. However, these have been useful for studies on the cell of origin for osteosarcoma development and gaining insight into new genes and pathways that influence osteosarcoma initiation, progression and the process of metastasis. Most of the reported models utilize Cre/LoxP mediated deletion of *Trp53* and/or *Rb1* (reviewed in [48]). A few other models have been created by transgenic overexpression of various oncogenes including *Fos* and SV40 large T antigen [49], or co-deletion of one copy of *Twist* and mutation of *Apc* [50]. Osteosarcoma can be induced with high efficiency in mice by osteoblast lineage specific deletion of the *Trp53* gene, although the latency and penetrance is greatly enhanced by co-deletion of both copies of the *Rb1* gene [51].These data are consistent with the centrality of *TP53* and *RB1* loss of function alterations in human osteosarcoma [23]. Mouse osteosarcoma induced by deletion of *Trp53* is genomically unstable, as is seen in human osteosarcoma [52].

Others have created mouse cell lines from spontaneously occurring osteosarcoma. In cases in which these osteosarcoma were induced on a uniform strain background, the osteosarcoma cells can be grown in syngeneic hosts by intravenous, subcutaneous, or orthotopic injections into the tibia (reviewed in [53]). Examples include the K12 and K7M2 derivative that is highly metastatic, and the Dunn along with its metastatic derivative LM8. These cell lines have been useful for testing the role of various components of the immune system on primary osteosarcoma tumor growth and osteosarcoma metastasis [54]. Such cell lines have also been used to create matched pairs of poorly and highly metastatic cell lines for study [53]. These have been useful for developing candidate genes and pathways that may regulate the process of metastasis, such as high-level expression of ezrin [28].

Quist *et al.* reported that osteosarcoma could be induced in mice via homozygous Cre/LoxP-mediated deletion of *Trp53* and *Rb1* in undifferentiated mesenchyme, pre-osteoblasts or infrequently cycling mature osteoblasts [6]. These data suggest that a variety of cell types could be targets for osteosarcoma development. This plasticity may also be the basis for the ready acquisition of new osteosarcoma phenotypes in patients, including chemo/radiotherapy resistance and the ability to readily colonize the lung.

From osteosarcoma mouse models, we have learned that osteosarcoma cells have a predilection to metastatic colonization of the lung, but, interestedly, have observed a metastatic potential spectrum across various cell lines. Intriguingly, in mouse models of Li-Fraumeni, the rate of lung metastasis is influenced by the nature of the p53 mutant allele used and when in the lifetime of the mouse Cre is used to induce mutation of the p53 gene [51]. The rate of spontaneous development of osteosarcoma and the frequency of lung metastasis is higher in mice heterozygous for the *Trp53^R272H^* allele than in mice heterozygous for the carry *Trp53^R270H^* allele [55].

Recently, a new mouse model of osteosarcoma was developed which utilizes the *Sleeping Beauty* (SB) transposon mutagenesis system [29]. In this project, a SB transposon vector designed to activate proto-oncogenes or inactivate tumor suppressor genes by insertional mutagenesis, was mobilized specifically in *Osx1+* cells using a tissue-specific mutagenesis approach. Mutagenesis by SB was able to induce osteosarcoma in otherwise wild type mice or accelerate osteosarcoma in mice with tissue-specific induction of the *Trp53^R270H^* allele. Transposon insertion mutations in specific regions of the genome are found recurrently in SB induced tumors and these regions are called “common insertion sites” or CIS [29]. CIS result from selection for insertions that occur within or near a cancer gene in the right position and the right orientation so as to give the cell a selective advantage and drive the development of a tumor. Thus, the identification of genes at or near CIS defines new candidate drivers of cancer development.

## 5. Conserved Drivers of Osteosarcoma

### 5.1. Genomic Alterations

It has been reported that genomic alterations are necessary for development of osteosarcoma in mouse models (*TP53*) and are accepted as being commonly shared in humans and dogs [13,29,56].

Highly chaotic karyotypes encompass a key feature that characterizes some mouse models used for osteosarcoma studies, and naturally occurring osteosarcoma in dogs, as well as in human patients. The average human osteosarcoma harbors roughly 30 coding alterations [23,57] while in dog osteosarcoma and mouse models, this figure is not known with certainty. Many of the candidate genes implicated in the pathogenesis and progression of osteosarcoma in people have also been characterized in the canine disease. Notable examples of these genes are *PTEN* (phosphatase and tensin homolog), *RB1* (retinoblastoma), and *TP53* (tumor protein 53), and *MET* (mesenchymal-epithelial transition factor).

Genetic alterations of the retinoblastoma susceptibility (*RB1*) gene have been implicated in the development and progression of osteosarcoma. In people, almost 70% of osteosarcomas have at least one *RB1* gene alteration and the percentage is similar in dog osteosarcomas [13]. We recently reported that an aberrant RB-E2F1 regulatory pathway is predictive of biological behavior [58]. Since our work, another group has reported that RB1 alterations may serve as a prognostic marker for the management of osteosarcoma patients [59].

Approximately 50% of human osteosarcomas have been reported to have somatic *TP53* deletions or point mutations detected using exon sequencing strategies [23]. However, a recent study that used whole genome sequencing of human osteosarcomas suggests that nearly all osteosarcoma tumors have p53 pathways lesions, which in many cases are translocations that break in the first intron of *TP53* gene [57]. Thus, it would appear that p53 pathway loss might be a requirement for osteosarcoma development in people. Clearly, germline *TP53* mutations, which cause Li-Fraumeni syndrome, predispose to osteosarcoma. However, it is unclear whether *TP53* mutations act as initiating mutations or progression mutations in sporadic osteosarcoma. In any case, p53 pathway alterations are common in genetically engineered mouse models as well as spontaneous canine and human osteosarcoma. Therefore, mouse models could be used to develop therapies that exploit p53 pathway alterations in patient osteosarcoma.

In humans and dogs, several oncogenes have been identified as possibly playing a role in osteosarcoma including *MET*, *FOS*, *IGF1R* (Insulin-Like Growth Factor 1 Receptor), *PVT1/MYC*, *RUNX2*, and *HER2*. Some of these changes involve copy number alterations. Consistent genome and chromosome copy number changes have been reported in canine and human osteosarcoma [17]. For instance, a high copy number gain of the *RUNX2* locus has been reported in both human and dog osteosarcoma [56].

Some of these genes, or the pathways they regulate, including the *PVT1/MYC* locus, *MET* signaling, and *IGF1R* have been implicated in genetically engineered mouse models of osteosarcoma [29,52].

Recurrent point mutations have been observed in osteosarcoma, but there are few beyond *RB1* and *TP53* that reach statistical significance. This is in part because too few samples have been sequenced. However, point mutations or deletions of several tumor suppressor genes, likely to be drivers of osteosarcomagenesis, were observed in a recent report of human osteosarcoma including *NF1*, *NF2* and *PTEN*, all of which were also recovered in a transposon-based screen for osteosarcoma in mice [29]. Overlap exists between other reported candidate human osteosarcoma genes after genome-wide copy number and whole exome sequencing were done [23], and also after transposon based screening in a mouse model including activation of *PI3KCA*, and *AKT1*, and inactivation of *ARID1A* [29].

### 5.2. Deregulation of Micrornas in Canine and Human Osteosarcoma

microRNAs (miRNAs) are small non coding RNAs that regulate more than 60% of the genome post transcriptionally. Several studies have identified deregulated miRNAs in osteosarcoma [60,61,62,63,64,65,66]. One of the earliest studies reported four differentially expressed miRNAs in a handful of osteosarcoma samples compared to controls [67]. Since these initial studies, additional research has shown that miRNA deregulation is potentially central to osteosarcoma development and progression [68]. Indeed, our group generated a Sarcoma MicroRNA Expression Database (S-MED) that represents 22 different sarcoma types, including osteosarcoma [69]. Through S-MED we found that osteosarcoma clustered separately from all other sarcoma types indicating the presence of potentially biologically important miRNAs. One of the unique miRNA signatures we found revealed the significant downregulation of around 50 miRNAs in the human 14q32 locus compared to the controls [65]. Most importantly, we have also shown a comparable decrease in expression of orthologous 14q32 miRNAs in canine osteosarcoma samples [65]. Interestingly we did not observe any copy number changes at the 14q32 locus, suggesting epigenetic changes at the locus. We confirmed that subset of these 50 miRNAs (miR-382, miR-369-5p, miR-544 and miR-134) could target the 3′ UTR of cMYC transcript. Additionally, overexpression of these miRNAs in osteosarcoma cells decreased the cMYC levels and induced apoptosis [65,70]. Also cMYC has been shown to transactivate a commonly known miRNA cluster, miR-17-92 [71]. We showed that restoration of the 14q32 miRNAs not only did decrease the cMYC levels but also significantly reduced the levels of mir-17-92 [70].

It was observed in other studies that miR-135b, -150, -370, -542-5p, -652, and -654 were highly expressed compared to osteoblasts [72,73,74]. Many of them play crucial roles in bone differentiation and key signaling pathways (miR-206 and -286) [75]. Additionally, miRNAs target *TP53*, which is an important tumor suppressor gene, was mutated in more than 60% osteosarcoma tumors [76]. One of the key miRNAs in the *TP53* pathway is miR-34, which is significantly downregulated in many osteosarcoma tumors that affects the cell cycle and proliferation [77,78,79].

The role of miRNAs in canine osteosarcoma remains to be fully elucidated. Since the discovery of miRNAs, there have been few studies that show a correlation between deregulation of miRNAs and canine osteosarcoma. A recent study investigated the role of miR-196a and its target Annexin V in human (143B, MG63) and canine (DAN) osteosarcoma cell lines to identify potential targets for new therapeutic agents in both the species [80]. They observed that miR-196a is downregulated in many of the canine osteosarcoma tumors compared to the normal bone, which is in contradiction with some earlier studies [66,81]. Further studies need to be performed on a larger cohort to establish that miR-196a is a potential therapeutic target and can impact multiple downstream targets in canine osteosarcoma.

A study led by Fenger *et al.*, sought to characterize miRNA expression in canine primary tumors among the major large breeds (greyhounds, rottweilers, golden retrievers and some mixed breeds) [43,82]. Using Nanominer software, they determined 189 miRNAs that were differentially expressed. Of these, miR-494 was highly expressed in all the breeds compared to normal canine osteoblasts.

As mentioned earlier, our group was among the first groups to observe a significant downregulation of around 50 miRNAs in the human 14q32 chromosomal region, which was highly conserved in the dog genome. Out of the 50 miRNAs, 2 miRNAs, miR-134 and mir-544, shared 100% conservation with the canine genome and mapped to the predicted synteny in canine osteosarcoma [14]. We have comprised a table of key miRNAs that play an important role in survival outcome and chemoresponse that can potentially lead to osteosarcoma diagnosis, subtyping and therapeutics in human and canine osteosarcoma (Table 1).

**Table 1:** miRNAs as potential markers for survival outcome and/ or chemosensitivity.

miRNAs Deregulated in OS	Expression Levels Compared to the Controls	Overall Function	References
miR-382	Down-regulated	Poor survival outcome and metastasis marker	[70,83]
miR-154	Down-regulated	Poor survival outcome	[70]
miR-33a	Up-regulated	Chemoresistance	[84]
miR-34c	Down-regulated	Chemoresistance	[85]

### 5.3. Epigenetic Changes in Osteosarcoma

Most forms of human cancer have changes in the epigenome compared to the normal cellular counterparts from which they are derived. Indeed, changes to the epigenome seem to be a ubiquitous feature of cancer. Epigenetic changes are revealed in alterations to the pattern of DNA methylation, histone modifications and nucleosome remodeling (reviewed in [24]). Osteosarcoma, like other forms of human cancer, seems to have many such epigenome changes compared to normal osteoblasts, the presumptive target cell for transformation. Several studies have analyzed the epigenome in a genome-wide manner, albeit with low numbers of cases [86,87]. These studies confirm that specific methylation events are heterogeneous among different cases of human osteosarcoma and that these differences may help explain differences in the clinical behavior of different osteosarcomas. These genome wide studies in human osteosarcoma have been restricted to the study of DNA methylation. Work on other chromatin modification remains to be done. The study of genome wide epigenetic changes has not been undertaken on a large scale in genetically engineered mouse models or canine osteosarcoma samples.

Many studies in osteosarcoma have focused on methylation of specific genes or gene regions. These studies suggest that certain genes and genetic pathways are subject to control by DNA methylation and attendant modifications to the histone code. Among these are alterations to the RB and p53 pathways. While these specific genes are not frequent targets of methylation based gene silencing, genes in these pathways have been reported as targets for pathogenic methylation. Specifically the *CDKN2A* locus, encoding the cyclin dependent kinase inhibitor p16^INK4a^ and the Mdm2 inhibitor p14^Arf^, has been reported [88,89]. Several genes that are targets of p53, or modulate p53 activity, have been shown to be methylated and silenced in osteosarcoma cell lines or xenograft tumors including *CDKN1A*, *HIC1* and *GADD45* [90,91,92]. Many other tumor suppressor genes are silenced by promoter hypermethylation in osteosarcoma cell lines including *RASSF1A*, *TIMP3*, DAPK1 and others (review in Morrow and Khanna, Critical Reviews in Oncogenesis, 2015). Methylation and silencing of tumor suppressive miRNA genes has also been observed as specific events in human osteosarcoma cell lines and primary tumors. Promoter hypomethyation, and probable overexpression, of oncogenes is less well studied in osteosarcoma, but several reports have been made suggesting such a mechanism, notably for the metastasis promoting gene *IRX1* and the growth factor gene *IGF2* [93,94]. Several candidate osteosarcoma oncogenes found in a transposon-based forward genetic screen in mice are also hypomethylated and overexpressed in human osteosarcoma compared to normal osteoblasts, including *SEMA4D*, *RAF1* and *PAK1* [29]. Some of these epigenetic changes, including aberrant repression and activation, are associated with loss of imprinting control at specific loci in osteosarcoma cells [95,96,97].

Genome wide studies of epigenetic alterations in human osteosarcoma need to incorporate many more samples before we learn to what extent they may explain molecular subtypes and clinical behavior of different cases. No such studies have been reported for canine osteosarcomas or mouse models either. When they are, they will allow cross-species comparisons that could help reveal the most meaningful alterations. Despite these limitations, it is apparent that osteosarcoma development is accompanied by changes to the epigenome that drive progression and confer specific cancer phenotypes. Published data does suggest that epigenome targeted therapies, including histone deacetylase inhibitors and DNA demethylating agents, can alter gene expression and inhibit osteosarcoma growth and/or metastasis [93,98]. Indeed, one study suggests that osteosarcoma cell lines can be reprogrammed using induced pluripotency genes and that the accompanying epigenetic changes reduce tumorigenic potential [99]. Taken together, these data suggest that modification of osteosarcoma epigenomes will be a useful therapeutic approach.

## 6. Advantages of A Multi-Species, Comparative Osteosarcoma Study Approach

Using comparative genetic studies in multiple organisms, it is possible to understand more about the inherited and somatically acquired genetic alterations that are informative of osteosarcoma risk and clinical behavior, especially metastasis. It is reasonable that genetic alterations that are present in human, dog, and mouse osteosarcomas represent the most likely true drivers of osteosarcoma progression, based on the concept of convergent evolution. Singly, mouse models offer opportunities to discover new genes and pathways important in disease initiation and progression. Dogs bring many other advantages to the table. Notably, since they show remarkable intra-breed homogeneity, which, together with noticeable interbreed heterogeneity, the dog offers distinctive opportunities to understand the genetics underlying osteosarcoma. Taken all together, an interspecies approach offers a possibly broader view and understanding of osteosarcoma since, for example, what is found to be altered at the genetic level in one species may be epigenetically silenced altered in another, but would be hard to pick out based on changes to gene expression alone.

Findings from mouse models and naturally occurring osteosarcoma in dogs need to be translated into therapeutic approaches in a clinically relevant model. Advantages of clinical trials in dogs are numerous and include that there are many more cases of canine osteosarcoma than human osteosarcoma [100,101]. In addition, disease progression in dogs, even with standard of care, is rapid so that assessments of improvement can be made in comparatively short periods of time [102].

It has been shown that histological response is not a good predictor (St. Jude, CTOS 20th Annual Meeting) in human osteosarcoma patients, and so there is an urgent need for informative biomarkers. The 14q32 locus is a new and promising marker, which was discovered by our group through a comparative species approach [65].

## 7. Conclusions

In more than 70% of osteosarcoma patients, current standard-of-care therapies ultimately fail to prevent relapse and metastasis. This dismal outcome has not improved over the past three decades, indicating a desperate need for novel drugs and treatment strategies. As osteosarcoma is genetically heterogeneous, successful therapies will need to target conserved pathobiology. In this review, we discussed the presence of multiple conserved signaling mechanisms as well as some of the approaches for understanding osteosarcoma pathobiology. Recent studies highlight the role of microRNAs in regulation of these conserved signaling pathways. In addition, we have just begun to understand the roles of the regulatory small RNAs and long-noncoding RNAs (lncRNAs). Further, intricacy in gene regulations does not end with identification of regulatory RNAs and its interacting partners. Molecular intricacy is also heavily dependent on our understanding of the implications of other layers of gene regulation such as role for competing endogenous RNAs along with pseudogenes, circular RNAs and lncRNAs. Acquiring such breadth and depth of knowledge is critical for developing therapies that will prevent bypass mechanisms of resistance to therapies. Moreover, cell non-autonomous functions of secreted factors such as exosomes are currently being investigated. In addition, emerging concepts of immune regulation by cancer and recent advancements in immunotherapy hold promise for treating and better outcomes in osteosarcoma.
